# Physics-Embedded Machine Learning Model for Phase Equilibrium Prediction in Multicomponent Systems

**DOI:** 10.1021/acs.jcim.5c01804

**Published:** 2025-09-22

**Authors:** Yue Yang, Shiang-Tai Lin

**Affiliations:** Department of Chemical Engineering, National Taiwan University, Taipei 106319, Taiwan

## Abstract

We present TeNNet-SAC (**T**hermodynamics-**e**mbedded **N**eural **Net**work for **S**egment **A**ctivity **C**oefficient) model, a novel machine learning framework for predicting activity coefficients in liquid mixtures using only the SMILES representations of the constituent molecules. Inspired by the quantum chemistry-based COSMO-SAC model, TeNNet-SAC evaluates activity coefficients by summing contributions from molecular surface segments. The model comprises three core components: (1) a σ-profile predictor, which generates molecular fingerprints (i.e., surface segment charge histogram or σ-profile) directly from SMILES; (2) a geometry predictor, which estimates molecular volume and surface area from SMILES; and (3) a Γ predictor, which computes the activity coefficients of surface segments in solution. The σ-profile and geometry predictors are trained on 39,745 quantum solvation calculations. The Γ predictor is initially pretrained on one million synthetic data points to capture physically consistent behavior and is subsequently fine-tuned end-to-end using experimental activity coefficient data to improve predictive accuracy. The base TeNNet-SAC model achieves accuracy comparable to COSMO-SAC, while the fine-tuned version consistently outperforms COSMO-SAC across benchmark systems. By treating segment activity coefficients as intermediate variables, TeNNet-SAC naturally generalizes to multicomponent mixtures and satisfies thermodynamic consistency, offering a robust and scalable solution for activity coefficient prediction.

## Graphical abstract

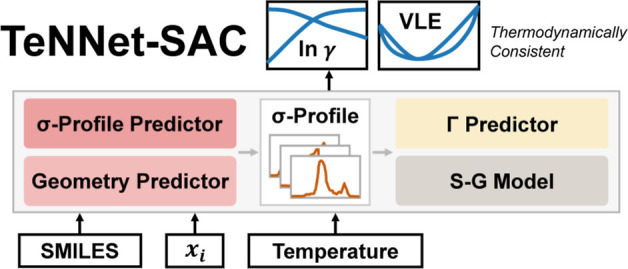

## 1 Introduction

Activity coefficients are fundamental to a wide range of chemical engineering applications, particularly in modeling fluid-phase equilibria and designing separation processes such as distillation,^1,2^ liquid–liquid extraction,^3,4^ and crystallization.^5,6^ However, experimental determination of activity coefficients is often labor-intensive, costly,^7^ and, in some cases, hazardous due to chemical toxicity or the need to operate under extreme temperature and pressure conditions.^8,9^ As a result, experimental data remain limited. For example, it has been estimated that only a minute fraction (approximately 6.9 × 10^–5^) of all possible binary mixtures have recorded data in the Dortmund Data Bank (DDB).^7,10^ These challenges have created a pressing need for accurate, efficient, and broadly applicable predictive models for activity coefficients.

Several traditional physical modeling approaches have been developed to address this challenge. These include quantitative structure–property relationship (QSPR) models,^11–13^ quantum chemical–statistical mechanical (SM) models,^14,15^ and group contribution (GC) methods.^16,17^ While these methods offer a certain degree of predictive capability, they often suffer from inherent limitations in flexibility, transferability, and accuracy when applied to chemically diverse systems.^18–20^

Recent advances in machine learning (ML) have spurred the development of data-driven models^21–25^ for estimating activity coefficients in mixtures, often outperforming well-established physical models in predictive accuracy.^21–23,25^ These developments underscore the considerable potential of ML in chemical thermodynamics. However, several fundamental challenges remain.

First, many ML models may not rigorously satisfy physical constraints such as the Gibbs–Duhem consistency relation,^26^ which is essential for generating consistent predictions of phase diagrams from the excess Gibbs free energy or from the activity coefficient (composition derivative of the excess Gibbs free energy).^27,28^ One possible solution is to embed ML within traditional thermodynamic frameworks that are inherently consistent. For example, ML can be used to predict NRTL^29^ parameters, as in the SPT-NRTL model.^22^ While this strategy ensures Gibbs–Duhem compliance, it also inherits the limitations of the underlying physical model, including limited flexibility and generalizability. An alternative approach is to integrate physical knowledge directly into the ML architecture through soft or hard constraints.^23,26,30,31^ In soft constraint methods, thermodynamic consistency is enforced by adding appropriate terms to the loss function, allowing the model to learn the constraints implicitly.^26^ A well-known example of this strategy is physics-informed neural networks (PINNs),^30^ in which differential equations are incorporated into the loss function as residual terms. However, these approaches provide no guarantee that the predictions will strictly obey the underlying physical laws. Hard constraint methods, such as HANNA^23^ and GE-GNN,^24^ explicitly embed the Gibbs–Duhem relation into the model structure. While these architectures improve physical interpretability and compliance, they are currently applicable only to binary mixtures.

The second challenge of ML models for activity coefficient prediction is their generalization to mixtures with three or more components. Most existing models are designed for binary systems^23,24^ or rely on binary interaction parameters,^22,25,32^ limiting their applicability to true multicomponent mixtures.

The third challenge is the need for large amounts of high-quality data. ML models typically require large training datasets to perform well, which presents a significant bottleneck given the scarcity of high-quality experimental thermodynamic data.^33,34^ Developing models that generalize effectively under data-scarce conditions remains a major challenge. Moreover, even models that perform well within the distribution of the training data often struggle with extrapolation to new chemical systems, unless guided by strong theoretical principles.^35,36^ One potential solution is to pretrain ML models on synthetic or computationally generated data^23^ and subsequently fine-tune them using experimental measurements. However, fine-tuning with limited experimental data frequently results in catastrophic degradation of model performance.^37,38^

In this work, we propose a composite machine learning model designed to address the aforementioned challenges. The Thermodynamics-embedded Neural Network for Segment Activity Coefficient (TeNNet-SAC), inspired by the quantum chemistry-based COSMO-SAC framework, predicts molecular activity coefficients by summing contributions from surface segments. The model includes a module that estimates the σ-profiles (histograms of surface segment charge distributions when a molecule is embedded in a perfect conductor) directly from the SMILES^39^ representations of the constituent molecules. A second module learns the relationship between segment charges and segment activity coefficients using a large synthetic dataset generated from the COSMO-SAC model.^15^ This module is hard-constrained to ensure thermodynamic consistency in the predicted segment activity coefficients. As a result, TeNNet-SAC is naturally applicable to multicomponent systems and rigorously satisfies thermodynamic consistency. The modular architecture of TeNNet-SAC provides significant flexibility for model development. For example, the SMILES-based σ-profile predictor can be replaced with computationally derived σ-profiles when available. Moreover, synthetic data generated by applying COSMO-SAC to artificial σ-profiles offer a physically grounded pretraining strategy, enabling robust fine-tuning even under data-scarce conditions.

## 2 Model Development and Training Strategy

This section presents the development and training strategy of the proposed TeNNet-SAC model, which is designed to predict molecular activity coefficients using only the SMILES representations of the mixture components as input. The model development comprises three main stages: (i) training a σ-profile predictor and a geometry predictor (for estimating molecular surface areas and volumes) from SMILES strings; (ii) training a segment activity coefficient (Γ) predictor using artificial σ-profiles; and (iii) end-to-end fine-tuning the integrated model using a small set of experimental molecular activity coefficients. The theoretical foundations and overall workflow are presented in Section 2.1. The datasets used in each stage are introduced in Section 2.2, and the model architectures are described in Section 2.3. Training strategies, evaluation metrics, and implementation details are provided in the Supporting Information.

### 2.1 Model Workflow and Theoretical Foundations

The TeNNet-SAC model follows a similar mechanism of the statistical mechanical COSMO-SAC model for predicting activity coefficients in a multicomponent liquid mixtures. In COSMO-SAC, the molecular activity coefficient of component *i* in a solution *S*, denoted as γ_*i*/*S*_, is computed as the sum of two contributions: a residual term accounting for intermolecular interactions, and a combinatorial term that captures differences in molecular size and shape

1 $$\ln\gamma_{i/S}=\ln\gamma_{i/S}^{res}+\ln\gamma_{i/S}^{comb}$$

The combinatorial contribution is evaluated using the Staverman–Guggenheim formalism^40,41^

2 $$\ln\gamma_{i/S}^{comb}=\ln\frac{\phi_{i}}{x_{i}}+\frac{z}{2}q_{i}\ln\frac{\theta_{i}}{\phi_{i}}+l_{i}-\frac{\phi_{i}}{x_{i}}\sum_{j}x_{j}l_{j}$$

where *x*_*i*_ is the mole fraction of component *i*, *θ*_*i*_ = (*x*_*i*_ *q*_*i*_)/(∑_*j*_ *x*_*j*_ *q*_*j*_) is surface fraction of component *i*, *ϕ*_*i*_ = (*x*_*i*_ *r*_*i*_)/(∑_*j*_ *x*_*j*_ *r*_*j*_) is the volume fraction, *l*_*i*_ = (*z*/2)­[(*r*_*i*_ – *q*_*i*_) – (*r*_*i*_ – 1)] and *z* = 10 is the coordination number, *r*_*i*_ = *V*_*i*_/*V*_ref_ and *q*_*i*_ = *A*_*i*_/*A*_ref_ are normalized volume and surface area of *i*, *V*_ref_ and *A*_ref_ are normalization constants.

The residual term is determined with consideration of molecular interactions through surface contacts. The interactions are evaluated based on the surface screening charges when the molecule is surrounded in a perfect conductor. The distribution of the surface screening charges, known as the σ-profile, representing the response of the molecule to an external dielectric medium is the key to the evaluation of activity coefficient. To facilitate the calculation, the molecular surface is dissected into equal-sized segments, each possessing some charge density. Therefore, in a mixture the probability of finding a segment with charge density σ is determined as

3 $$p_{s}\left(\sigma \right)=\frac{\sum_{i}x_{i}p_{i}\left(\sigma \right)A_{i}}{\sum_{i}x_{i}A_{i}}$$

The core of the COSMO-SAC model is the calculation of segment activity coefficient Γ_*k*_(σ) in the solution described by the σ-profile

4 $$\ln\Gamma_{k}\left(\sigma_{m}\right)=-\ln\left\{\sum_{\sigma_{n}}p_{k}\left(\sigma_{n}\right)\Gamma_{k}\left(\sigma_{n}\right)\exp\left[\frac{-\Delta W\left(\sigma_{m},\sigma_{n}\right)}{kT}\right]\right\}$$

where subscript *k* can be either the mixture solution *S* or pure component *i*, Δ*W*(σ_*m*_,σ_*n*_) denotes the interaction energy between a pair of segments with charge densities σ_*m*_ and σ_*n*_, respectively. The residual contribution to the activity coefficient, ln γ_*i*/*S*_^res^, is then determined by a weighted summation over the difference in logarithmic segment activity coefficients ln Γ_*k*_(σ) between the solution *S* and pure component *i*

5 $$\ln\gamma_{i/S}^{res}=n_{i}\sum_{\sigma_{m}}p_{i}\left(\sigma_{m}\right)\left[\ln\Gamma_{S}\left(\sigma_{m}\right)-\ln\Gamma_{i}\left(\sigma_{m}\right)\right]$$

where $n_{i}=\frac{A_{i}}{a_{eff}}$ is the number of effective surface segment, *A*_*i*_ is the surface area and *a*_eff_ = 5.8447 Å^2^ is a standard segment surface area.^42^

To enable this procedure through machine learning, we developed a hard-constrained neural network model,^23^ referred to as the Γ predictor, that predicts segment activity coefficients from σ-profiles and temperature, while ensuring thermodynamic consistency. In addition, to relieve the burden of computationally expensive quantum chemical calculations, two additional machine learning models, the σ-profile predictor and the geometry predictor, are developed to predict molecular σ-profiles and geometry directly from SMILES strings. A few ML models have been developed for this purpose.^43–46^ Here we adopted pretrained SMILES encoders, SMI-TED298M,^47^ and ChemBERTa-2,^48^ to convert SMILES strings into dense molecular embeddings. These embeddings are used as inputs for two downstream predictors trained separately: (i) the σ-profile predictor, which outputs a σ-profile vector representation, and (ii) the geometry predictor, which predicts the molecular surface area and volume.

By combining these single-task components, the complete modeling workflow is established, as illustrated in Figure 1. The first pathway transforms SMILES strings into molecular σ-profiles using the σ-profile predictor and the geometry predictor. These surface area-weighted σ-profiles are then used by the Γ predictor to estimate segment activity coefficients, which in turn yield the residual activity coefficients. The second pathway directly derives the molecular surface area and volume from SMILES strings using the geometer predictor, and employs these geometric features to compute the combinatorial contribution via the Staverman–Guggenheim formalism. The resulting composite architecture is referred to as the base model.

**Figure 1:**
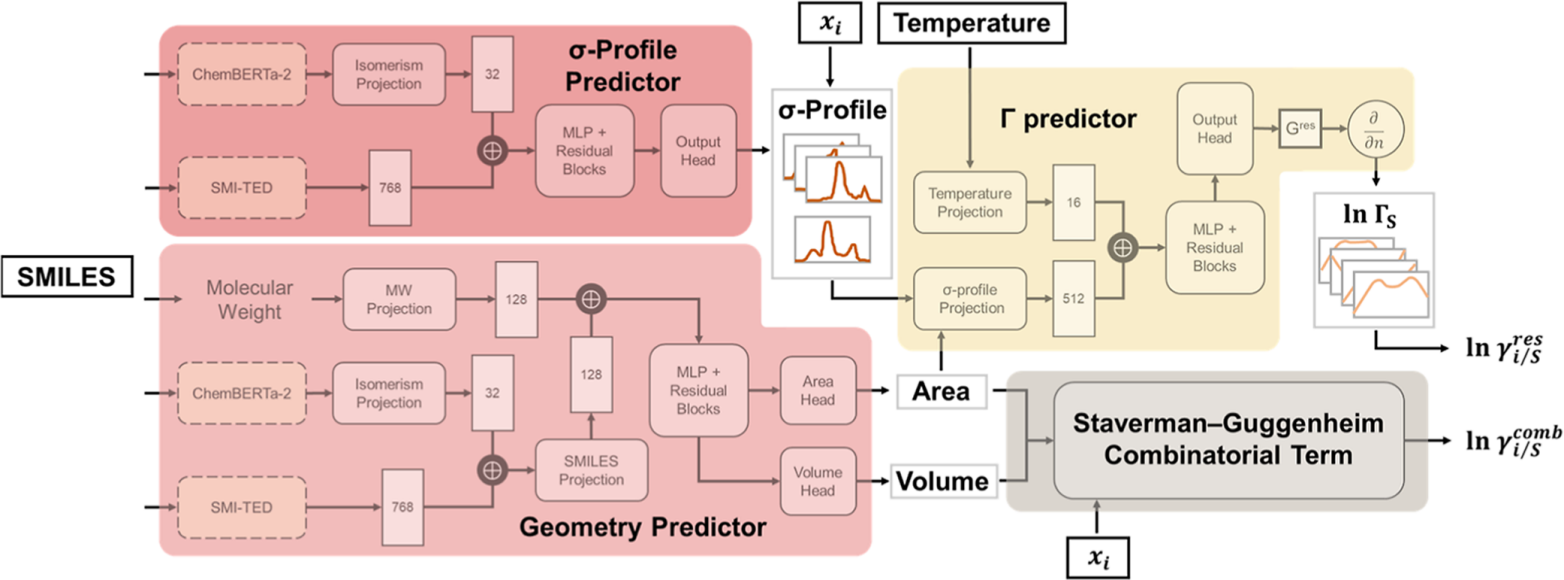
Architecture of the TeNNet-SAC model. It consists of four components: three ML models (σ-profile predictor, geometry predictor, and Γ predictor) and one physical model (the Staverman–Guggenheim model). This end-to-end framew requires only SMILES strings, temperature, and composition as input to predict molecular properties.

To effectively develop this modular architecture, the three components of the base model are initially trained independently. In the first stage, the σ-profile and geometry predictors are pretrained on large-scale datasets derived from quantum chemical calculations. At the same time, the Γ predictor is trained using synthetic data, which are generated from artificially constructed σ-profiles that do not correspond to any real molecules. The corresponding segment activity coefficients are calculated using eq 4, enabling the Γ predictor to learn physically consistent behavior without relying on computational expensive quantum chemically generated σ-profiles or scarce experimental data.

In the second stage, we perform end-to-end fine-tuning using a limited set of VLE-derived experimental activity coefficient data. During this process, only the parameters of the Γ predictor’s output head are updated, while the σ-profile and geometry predictors remain frozen. This training strategy enables the Γ predictor to align with experimental observations and improve prediction accuracy, while preserving the modularity and reusability of the upstream components. The base model training and fine-tuning procedures follow standard supervised learning practices. Details of the hyperparameters, loss functions, and convergence settings are provided in Section S3 of the Supporting Information.

### 2.2 Data Sets and Data Preprocessing

Three datasets were constructed to train and validate different components of the model: (i) quantum-chemically computed σ-profiles and molecular geometries (*D*_QC_), (ii) artificial σ-profiles paired with corresponding COSMO-SAC-derived segment activity coefficients (*D*_SAC_), and (iii) experimental molecular activity coefficients (*D*_EXP_).

#### 2.2.1 SMILES with Computed σ-Profiles, Areas, and Volumes (*D*_QC_)

A total of 39,745 neutral molecules were collected to construct the *D*_QC_ data set. Among them, 1,429 molecules were sourced from the VT-2005 database^49^ after excluding two mixture entries from the original 1,431 compounds. An additional 19,010 molecules were taken from a previous study evaluating the PR + COSMOSAC equation of state for vapor pressure prediction,^50^ where molecules exhibiting extreme surface charge densities (beyond ±0.025 e/Å^2^) were removed from the original set of 19,081. The remaining components were randomly selected from PubChem,^51^ subject to the constraints that their net molecular charges are zero and their molecular weights are less than 500 Da. Duplicate molecules across different sources were identified and removed to ensure that all entries in the dataset are unique.

For the generation of molecular σ-profiles, COSMO-based quantum chemical calculations were performed using the Amsterdam Modeling Suite (AMS).^52^ Geometry optimizations were carried out at the GGA Becke–Perdew (BP) level^53,54^ of theory with a TZP basis set, incorporating scalar relativistic corrections via the ZORA method.^55^ Following optimization, COSMO single-point calculations were conducted to obtain the screening charge densities. The COSMO-SAC charge-averaging procedure^15^ was applied to derive the molecular σ-profiles. The molecular surface area and volume were directly extracted from the COSMO calculation outputs.

The corresponding molecular SMILES strings were generated directly from the quantum-mechanically optimized structures using Open Babel^56^ and RDKit^57^ with isomeric information, including stereochemical descriptors for cis/trans and chiral centers.

The *D*_QC_ dataset was used to train both the σ-profile predictor and the geometry predictor, as it contains quantum-chemically computed descriptors required by these models. Since these components produce critical inputs for downstream components, maximizing training coverage was favored. Therefore, we adopted a 90%/10% train/validation split without reserving a dedicated test set for these two models. Additionally, to evaluate generalization performance, we trained identical models with the same settings using an 80%/10%/10% train/validation/test split. The test results reported in Section 3.1 are based on this alternative split.

#### 2.2.2 Synthetic σ Profiles with Segment Activity Coefficients (*D*_SAC_)

To construct the *D*_SAC_ dataset, artificial σ-profiles were generated to serve as inputs for training the Γ predictor. A total of one million artificial σ-profiles were created by randomly superimposing multiple Gaussian distributions with randomly assigned heights, widths, and positions, producing profiles similar in appearance to those in Figure 2. The initial profile *p*(σ_*j*_) was generated as shown in eq 6. The final form used in this work is the scaled profile *p̅*(σ_*j*_), which is truncated and normalized to match an area drawn from a uniform random distribution.

6 $$p\left(\sigma_{j}\right)=\sum_{m=1}^{3}\sum_{i=1}^{I}A_{mi}\exp\left(-\frac{{\left(\sigma_{j}-\sigma_{mi}\right)}^{2}}{2\delta_{mi}^{2}}\right)$$

where *A*_*mi*_, σ_*mi*_, and δ_*mi*_ are random numbers. Detailed parameter settings can be found in Section S1 of the Supporting Information.

**Figure 2:**
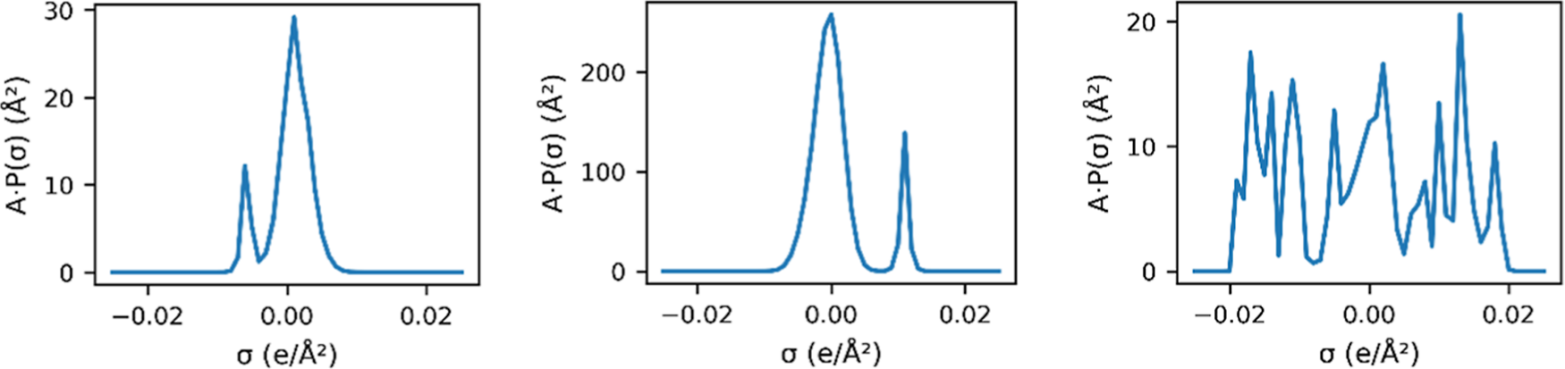
Illustration of three artificial σ-profiles generated by randomly superimposing multiple Gaussian functions, used for training the Γ predictor.

Each artificial σ-profile was paired with a randomly selected temperature between 200 and 800 K. The corresponding segment activity coefficients were then calculated using eq 4, treating each artificial σ-profile as if it represented a real molecule or a mixture. In this dataset, the input features are the artificial σ-profiles and temperatures, while the prediction targets are the corresponding segment activity coefficients (ln Γ). This synthetic generation approach ensures that the model learns the underlying principles of the COSMO-SAC formalism, rather than simply memorizing the statistical patterns observed in real σ-profiles. For training the Γ predictor on the *D*_SAC_ data set, we adopted a standard 80%/10%/10% split to ensure consistent evaluation.

In this work, the COSMO-SAC 2002 was adopted^15^ with model parameters optimized for the Amsterdam density functional (ADF) software package.^42^ A complete list of parameter values is provided in Section S7.2 of the Supporting Information.

#### 2.2.3 Experimental Molecular Activity Coefficients (*D*_EXP_)

The *D*_EXP_ dataset was constructed from experimental vapor–liquid equilibrium (VLE) data curated from the previous study by Chen and Lin.^58^ To enhance data reliability and consistency, all entries were evaluated using the built-in data quality scoring system implemented in Aspen Plus.^59^ The overall quality score, ranging from 0.1 to 1, is computed based on a series of consistency and reliability tests (see Section S2 of Supporting Information for details). Only data points with scores above 0.25 were retained for model training.

The final data set comprises a total of 397 unique binary systems, spanning 34,371 individual data points. The experimental *P–x–y* data are converted to the activity coefficients according to

7 $$\gamma_{i/S}=\frac{py_{i}}{p_{i}^{sat}x_{i}}$$

where the needed vapor pressure were estimated using the Wagner25 Equation^60^

8 $$\ln\frac{p^{sat}}{p_{c}}=\frac{1}{T_{r}}\left[C_{1}\left(1-T_{r}\right)+C_{2}{\left(1-T_{r}\right)}^{1.5}+C_{3}{\left(1-T_{r}\right)}^{2.5}+C_{4}{\left(1-T_{r}\right)}^{5}\right]$$

In this dataset, the input features consist of the SMILES strings of each component, the system temperature, and the mole fraction composition. The prediction targets are the corresponding molecular activity coefficients (ln γ_*i*/*S*_) for each component.

For end-to-end fine-tuning, the *D*_EXP_ data set was employed. To prevent data leakage, all entries associated with the same binary component pair were grouped together and assigned to the same data split. To ensure chemical diversity during training, the systems were roughly categorized into three groups based on their SMILES strings: (1) systems containing hydrogen-bonding compounds (HB); (2) systems containing N, O, F, or S atoms but lacking hydrogen-bonding functionality (polar, no HB); and (3) systems without these heteroatoms, generally considered nonpolar (weak/nonpolar). The data were then split into a 70%/20%/10% ratio for training, validation, and testing, while preserving the original category distribution across each split. This partitioning ensured chemical diversity and minimized category bias during model evaluation.

### 2.3 Architecture of Machine Learning Models

#### 2.3.1 The Pretrained SMILES Encoders

To encode SMILES strings into dense molecular embeddings, we employ two pretrained language models: SMI-TED289M^47^ and ChemBERTa-2 77M-MLM.^48^ SMI-TED289M is a deep bidirectional transformer architecture with 289 million parameters, pretrained on a curated corpus of 91 million unique SMILES strings from the PubChem database.^51^ It uses a combination of masked language modeling (MLM) and a SMILES reconstruction objective to learn meaningful molecular representations. Despite not being explicitly trained on quantum chemical properties, downstream studies^47,61^ have shown that the embeddings produced by SMI-TED enable accurate predictions of properties such as dipole moments, polarizabilities, and HOMO–LUMO gaps.

Given that molecular σ-profiles are derived from quantum chemical calculations, we selected the SMI-TED289M encoder as our primary feature extractor to capture important molecular features for σ-profile and geometries prediction. However, one limitation arising from the pretraining pipeline is its inability to process isomeric SMILES: stereochemical notations (e.g., @, /, \) are not effectively captured due to nonisomeric canonicalization applied during pretraining, which strips stereochemical information to ensure SMILES consistency. To mitigate this, we incorporated ChemBERTa-2 77M-MLM, a RoBERTa-based transformer model^62^ pretrained on 77 million SMILES using the MLM objective. To enhance the model’s sensitivity to different SMILES representations of the same molecule, we extracted mean-pooled embeddings from ChemBERTa-2’s final hidden layer, resulting in fixed-length 384-dimensional vectors that serve as auxiliary features to capture stereochemical and structural subtleties.

In our workflow, SMILES strings are first canonicalized using RDKit^57^ to remove stereochemistry before being input to the SMI-TED289M encoder, which yields a 768-dimensional embedding used as the primary molecular representation. In parallel, the original SMILES strings are canonicalized while preserving stereochemistry and passed to ChemBERTa-2, generating 384-dimensional embeddings that serve as auxiliary stereochemistry-aware descriptors. Together, these fused embeddings enable the downstream model to robustly predict molecular σ-profiles and geometries without sacrificing stereochemical information.

#### 2.3.2 σ-Profile Predictor and Geometry Predictor

The σ-profile represents the distribution of molecular surface charge density and serves as the primary molecular descriptor for predicting residual activity coefficients in this work. The surface segment charge density spans the range −0.025 to 0.025 e/Å^2^, a convention widely adopted in the literature and generally sufficient for describing neutral species. In practice, the σ-profile is discretized into a 51-dimensional vector.

To predict these molecular descriptors from SMILES input, we developed two separate models: a σ-profile predictor, which estimates the 51-dimensional σ-profile, and a geometry predictor, which outputs the molecular surface area and volume. Both models take as input a fusion of pretrained molecular embeddings from SMI-TED289M and ChemBERTa-2 77M-MLM. Additionally, the geometry predictor incorporates the molecular weight computed from the SMILES string.

The architecture of the σ-profile predictor is illustrated in Figure 3. It primarily relies on the 768-dimensional embeddings from SMI-TED289M, which provide a learned representation of the input SMILES. To incorporate stereochemical information, the 384-dimensional ChemBERTa-2 embeddings are passed through a projection module, reducing them to 32 dimensions. These compressed embeddings are concatenated with the SMI-TED289M embeddings, forming a combined 800-dimensional vector. This fused input is processed through a feedforward backbone with residual blocks, followed by a multilayer prediction head. The final output layer uses ReLU activation to ensure that all predicted values are non-negative, in accordance with the physical interpretation of σ-profiles as surface segment densities. The sum of all segment values is computed and used to normalize the output, ensuring that the final σ-profile is normalized. It is noteworthy that molecular charge neutrality is not enforced in the σ-profile predictor. Imposing charge-neutrality with soft or hard constraints in the σ-profile predictor and its influence on the predicted σ-profile and the molecular activity coefficients are discussed in Section S6 of the Supporting Information.

**Figure 3:**
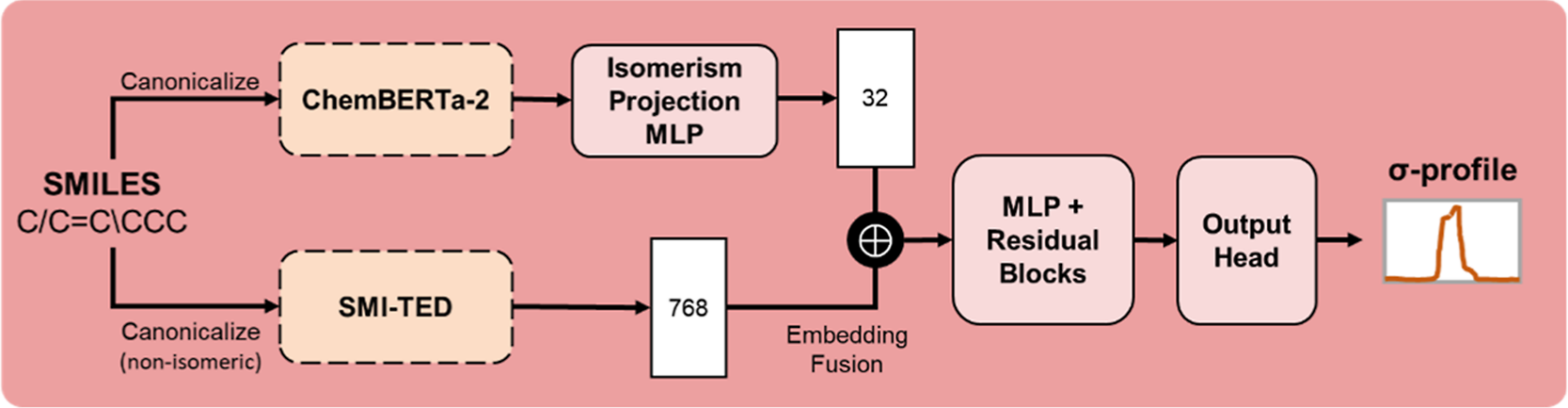
Architecture of the σ-profile predictor. Dashed-line blocks indicate frozen pretrained models. The model takes a SMILES string as input and outputs a 51-dimensional vector representing the σ-profile.

The architecture of the geometry predictor is illustrated in Figure 4. It shares the same embedding strategy: ChemBERTa-2 embeddings are compressed to 32 dimensions and concatenated with the 768-dimensional SMI-TED289M embeddings to yield a unified 800-dimensional vector. A difference is the inclusion of molecular weight as an additional input. This scalar is projected through a feedforward neural network into a 128-dimensional vector, which is then concatenated with the fused molecular embedding. The combined representation is passed through fully connected layers and a residual block. The final layer bifurcates into two output heads, each responsible for predicting a single scalar: molecular surface area and molecular volume.

**Figure 4:**
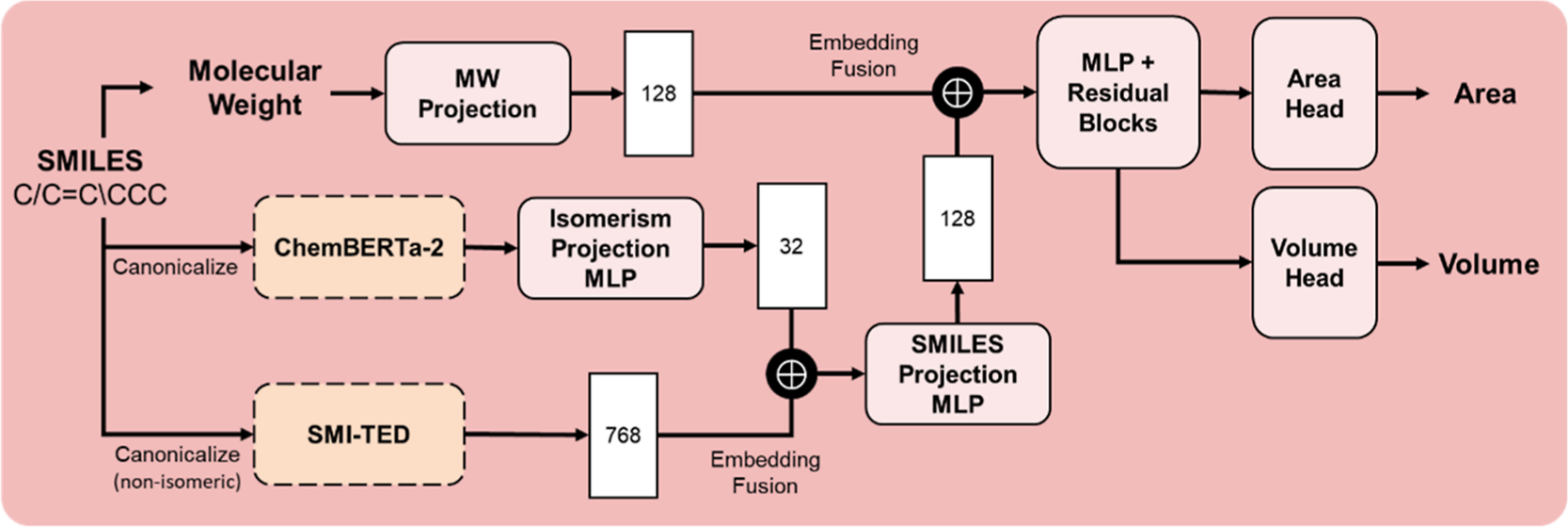
Architecture of the geometry predictor. Dashed-line blocks indicate frozen pretrained models. The model takes a SMILES string as input and predicts molecular surface area and volume.

Finally, the surface area-weighted σ-profile is obtained by multiplying the σ-profile from the σ-profile predictor with the molecular surface area from the geometry predictor. This molecular profile can be used in downstream models such as the Γ predictor developed in this work, or serve directly as input for COSMO-based methods like COSMO-SAC. Additionally, the predicted molecular area and volume can also be applied in eq 2 to compute combinatorial contributions to activity coefficients.

#### 2.3.3 Thermodynamics-Embedded Γ Predictor

To align with the physical framework of the COSMO-SAC model and approximate the behavior described by eq 4, a Γ predictor was designed to estimate segment activity coefficients based on a given σ-profile and temperature. Rather than explicitly computing the statistical-mechanical formulation, the predictor learns a functional mapping from molecular descriptors to segment activity coefficients. To ensure the output segment activity coefficients satisfying the Gibbs–Duhem thermodynamic consistency relation,^63^ the model first predicts the molar excess free energy and the segment activity coefficients are computed from the partial derivative with respect to the segmental σ-profile

9 $$\ln\Gamma_{k}\left(\sigma_{m}\right)=\frac{1}{a_{eff}}{\left[\frac{\partial A_{k}{\mathrm{G̲}}_{k}^{ex,res}}{\partial n_{k}\left(\sigma_{m}\right)}\right]}_{T,P,n_{k}\left(\sigma_{n\neq m}\right)}$$

where *n*_*k*_(σ_*m*_) = (*A*_*k*_ *p*_*k*_ (σ_*m*_)/*a*_eff_) denotes the number of surface segments for fluid *k* (can be a pure component or a mixture) of charge density σ_*m*_; *A*_*k*_ is the surface area of the fluid; Γ_*i*_(σ_*m*_) represents the activity coefficient of corresponding segment of charge density σ_*m*_. and ${\mathrm{G̲}}_{k}^{ex,res}$ is the residual component of the dimensionless molar excess free energy of the fluid. By incorporating this formulation, the predicted segment activity coefficients inherently satisfy thermodynamic consistency,^64^ ensuring that the resulting molecular activity coefficients also conform to fundamental thermodynamic principles (see Section S8 of the Supporting Information for the proof). A similar approach has been used in prior ML-based studies^23,31^ for binary mixtures. Here we generalize the calculation to multisegment systems. The σ-profile not only serves as a molecular descriptor,^65,66^ but also directly appears in the thermodynamic formulation of the model, giving it clear physical significance.

The architecture of the Γ predictor is illustrated in Figure 5. The model takes as input a σ-profile and a temperature. The σ-profile (51 dimensions) is first normalized and then passed through a multilayer perceptron to generate a 512-dimensional latent embedding. In parallel, the inverse temperature (1/*T*) is encoded into a 16-dimensional vector via a separate feedforward network. The two embeddings are concatenated and passed through a backbone composed of fully connected layers, batch normalization, and a residual block. The model outputs a scalar ${\mathrm{G̲}}_{i}^{ex,res}$ , corresponding to the molar excess free energy associated with the input σ-profile. To obtain the corresponding 51-dimensional segment activity coefficient vector, the gradient of ${\mathrm{G̲}}_{i}^{ex,res}$ with respect to the σ-profile is computed via automatic differentiation. This yields a differentiable and physically grounded representation of segment activity coefficients, consistent with eq 4. With segment activity coefficients defined for a given σ-profile, residual activity coefficients for all components in the system can then be computed using eq 5.

**Figure 5:**
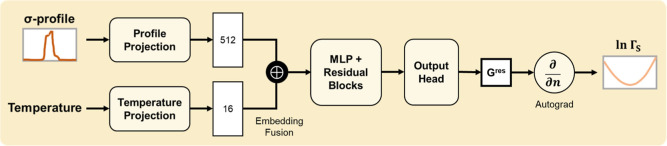
Model takes a σ-profile and temperature as input, and outputs the corresponding 51-dimensional segment activity coefficient vector. Thermodynamic consistency is enforced by ensuring that the output satisfies the Gibbs–Duhem equation through automatic differentiation.

It is noteworthy that replacing the SAC part of the COSMO-SAC model (eq 4) with a neural network model can enhance both computational efficiency and model flexibility. As a well-developed model, further improvements to COSMO-SAC typically involve introducing additional parameters, which increases model complexity and empiricism. However, as we will illustrate, the inherent flexibility of neural network models enables performance gains through fine-tuning as data quantity and quality improve. Moreover, extending the neural network model to more complicated mixtures, such as polymers, electrolytes, and weak acids may be more straightforward. We also benchmarked the computational efficiency of the Γ predictor against the SAC component of COSMO-SAC, and found that both approaches exhibit comparable runtimes on CPU. However, when executed on GPU, particularly with large batch sizes, the Γ predictor can be orders of magnitude faster. Detailed results are provided in Section S7.1 of the Supporting Information.

## 3 Results and Discussion

This section presents the results of our model in three key stages: (i) prediction of molecular descriptors, (ii) performance of the TeNNet-SAC base model compared to COSMO-SAC, and (iii) fine-tuning of TeNNet-SAC with experimental data to demonstrate practical applicability.

### 3.1 Prediction of Molecular σ-Profile from SMILES

This section evaluates the model’s ability to predict molecular σ-profiles from SMILES strings using two components: a σ-profile predictor and a geometry predictor. The model was evaluated on a test dataset excluded from training.

Figure 6 compares the predicted molecular surface areas and volumes with quantum chemically calculated values, showing strong correlations in both cases. Detailed training, validation, and testing metrics are provided in Section S5 of the Supporting Information. The surface area prediction is slightly more challenging than volume prediction (*R* ^2^ = 0.997 for surface area, and 0.999 for volume; % MAE = 1.24% for surface area, and 0.91% for volume). This is because exposed molecular surface area, and therefore the σ-profile, is more sensitive to the 3D molecular conformations, particularly in the case of long, flexible molecules. As SMILES representations do not explicitly encode 3D conformational information, such conformational variability cannot be fully captured, influencing the predictive accuracy for properties that are conformation-dependent. To mitigate this, we retain isomeric information in the SMILES representation to preserve as much structural detail as possible. Fortunately, for most common compounds, variations arising from cis–trans isomerism, chirality, or conformers do not lead to significant changes in the σ-profile, enabling the model to deliver reliable and practically useful predictions.

**Figure 6:**
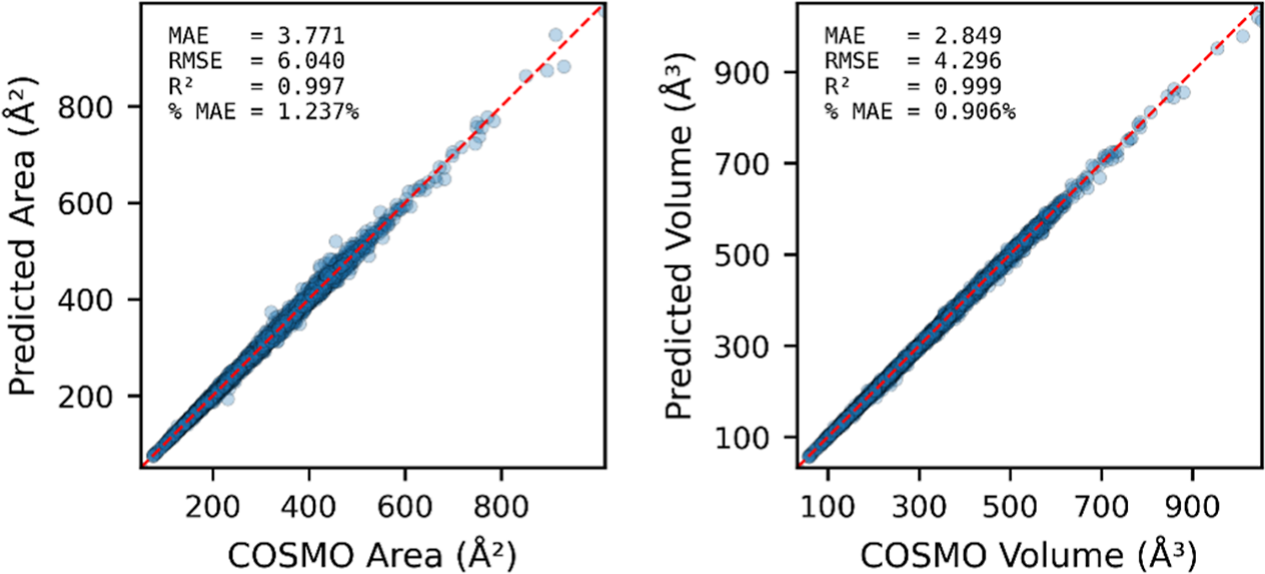
Scatter plots showing predicted area and volume versus COSMO values obtained from quantum chemical calculations.

Figure 7 illustrates the performance of σ-profile prediction. Detailed statistics for the training, validation, and testing sets are provided in Section S4 of the Supporting Information. The σ-profile predictor estimates the probability distribution of surface charge density, while the geometry predictor determines the surface area scaling of the profile. As shown in the figure, the average predicted σ-profile (blue line) aligns almost perfectly with the average reference profile from quantum chemical calculations (orange dashed line), indicating that the model is unbiased on average. The standard deviation of prediction errors (shaded light-blue area) is primarily concentrated in the nonpolar region (−0.005 to 0.005 e/Å^2^). This deviation is acceptable in practice, as minor fluctuations in the nonpolar region of a σ-profile do not significantly affect the computed segment activity coefficients. Figure 8 illustrates the predicted σ-profile with the QM calculated ones for three compounds (cycloheptene, 4-heptanol, and hexyl butanoate). The excellent agreement with the predicted σ-profile and the QM result supports direct use of the result in COSMO-SAC for activity coefficient calculations. In our framework, the predicted σ-profiles serve as informative features for downstream model, the Γ predictor.

**Figure 7:**
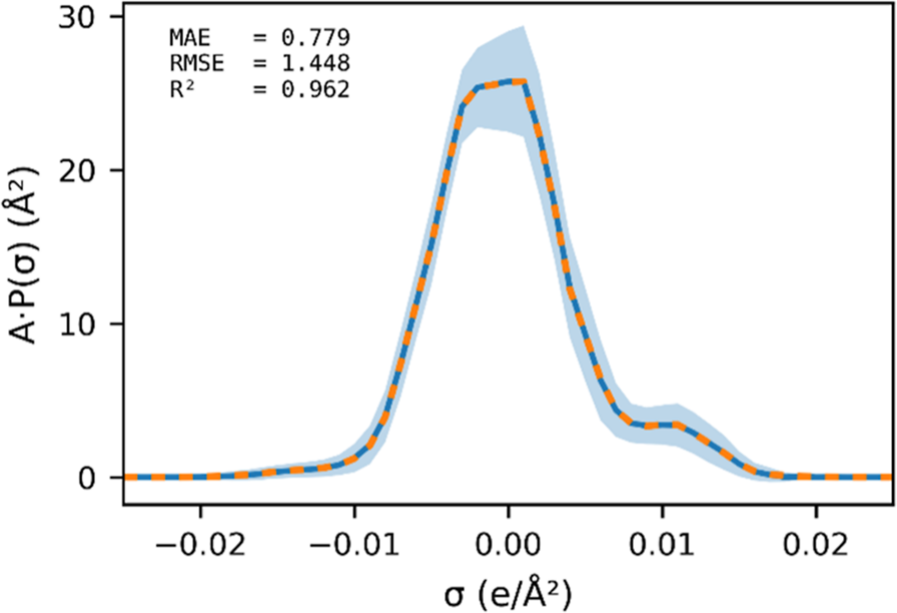
Average σ-profile of the test data set. The blue line represents the predicted profile; the orange dashed line shows the quantum chemistry-derived profile. The light-blue shaded area indicates the standard deviation of the prediction errors.

**Figure 8:**
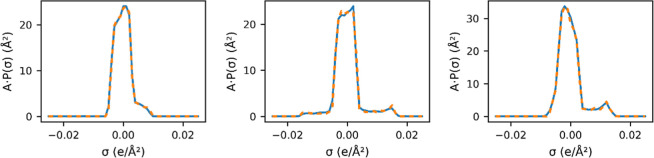
Examples of σ-profile predictions. From left to right are 1-methyl cycloheptene (CC1=CCCCCC1), 4-heptanol (CCCC­(O)­CCC) and hexyl butanoate (CCCCCCOC­(=O)­CCC). The blue line represents the predicted profile; the orange dashed line shows the quantum chemistry-derived profile.

### 3.2 Performance of the TeNNet-SAC Base Model

The TeNNet-SAC base model integrates the σ-profile predictor, a geometry predictor and a Γ predictor for molecular activity coefficient predictions (see Figure 1). It is noteworthy that the Γ-predictor in the base model is pretrained using synthetic data from the *D*_SAC_ dataset. The Γ-predictor did not “see” any real chemical data during training. Since segment activity coefficients are intermediate quantities within the COSMO-SAC, we assess the performance of the TeNNet-SAC base model by comparing the predicted molecular activity coefficients with those from the COSMO-SAC model. This end-to-end evaluation better reflects the practical relevance and predictive capability of the proposed framework.

Figure 9 compares the predicted ln γ values from the COSMO-SAC model with those from TeNNet-SAC base model but with different input sources. The left panel shows results from the TeNNet-SAC base model (this work), which predicts molecular features (σ-profiles, surface areas, and volumes) directly from SMILES representations, whereas the right panel shows results from the TeNNet-SAC model using quantum chemically derived σ-profiles, surface areas, and volumes as inputs. Each point represents a randomly sampled binary mixture from the VT-2005 database, at a randomly selected composition and temperature. A total of 100,000 such conditions were generated to comprehensively evaluate consistency across a broad chemical space. Using only SMILES strings as the input, the TeNNet-SAC based model (left panel) achieves an MAE of 0.065 and an MSE of 0.040. If QC calculated results are available, the agreement with the COSMO-SAC model can be improved to a lower MAE of 0.030 and MSE of 0.011. In practice, generating quantum chemically derived inputs requires substantial computational resources, making it impractical for rapid prediction tasks. The slightly higher inaccuracy from using the σ-profile generator can be effectively reduced during the fine-tuning step.

**Figure 9:**
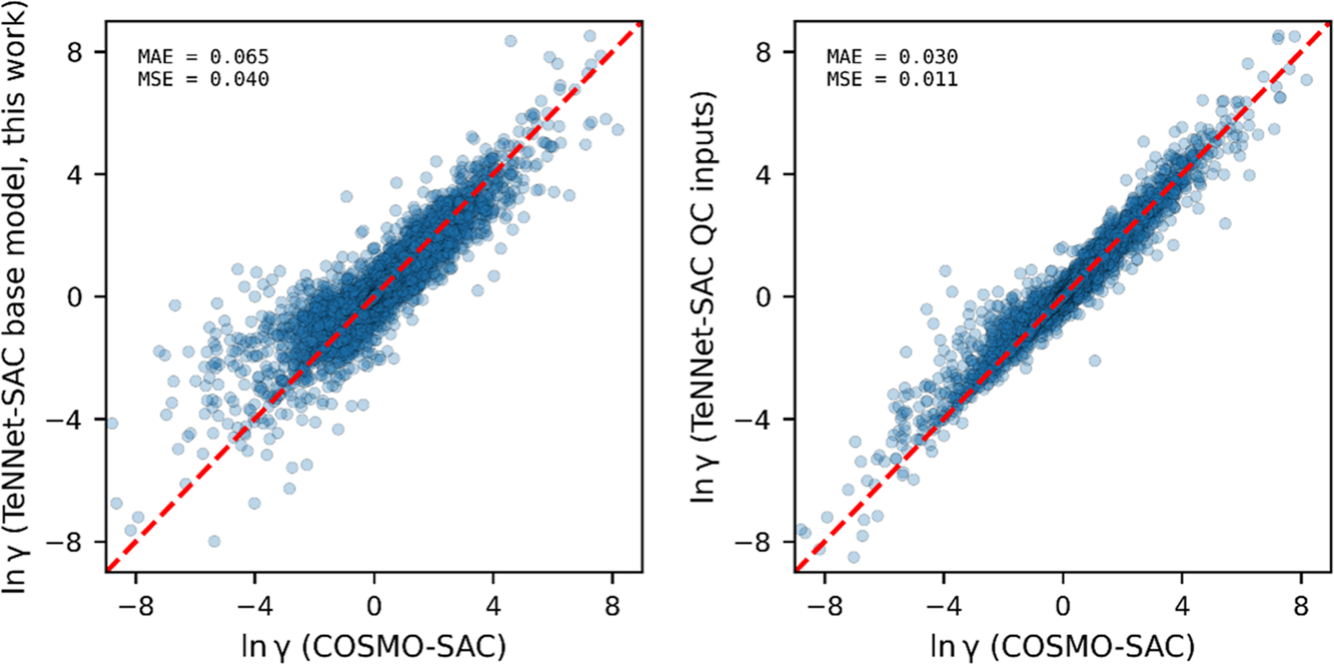
Comparison of predicted ln γ from COSMO-SAC with the TeNNet-SAC base model (this work) and the TeNNet-SAC model using QC-derived inputs for 100,000 randomly sampled binary systems from the VT-2005 database. The left panel shows results using SMILES-derived features, while the right panel uses quantum chemically derived σ-profiles, surface areas, and volumes as model inputs.

Figure 10 further presents representative predictions of excess Gibbs free energies, logarithmic activity coefficients, and isothermal vapor–liquid equilibrium diagrams for five selected binary systems. These systems cover three types of molecular polarity combinations: nonpolar–nonpolar, nonpolar–polar, and polar–polar. In all cases, the TeNNet-SAC base model successfully captures the characteristic trends predicted by COSMO-SAC.

**Figure 10:**
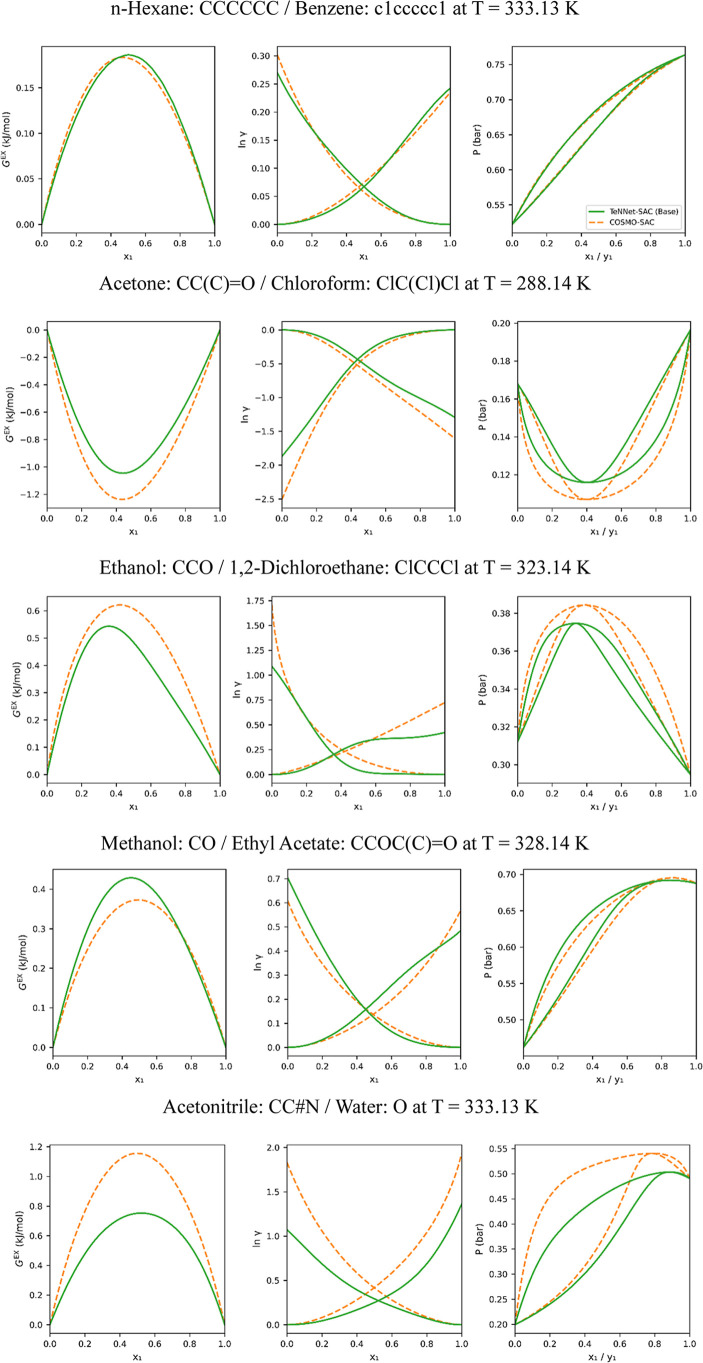
From left to right: predicted excess Gibbs free energies (*G* ^ex^), logarithmic activity coefficients (ln γ), and isothermal vapor–liquid equilibrium diagrams for binary systems. The base model (TeNNet-SAC) predictions are shown as blue lines, and COSMO-SAC results are represented by orange dashed lines.

It is important to emphasize that the Γ predictor was never trained on true molecular σ-profiles, nor was it directly trained to predict molecular activity coefficients. Although its upstream components (the σ-profile predictor and the geometry predictor) were trained on data from the VT-2005 dataset, this does not diminish the generalization capability of the Γ-predictor. On the contrary, the model’s strong alignment with COSMO-SAC across unseen mixtures demonstrates that it has learned the underlying principles of the COSMO-SAC algorithm rather than merely memorizing molecular properties.

### 3.3 Fine-Tuning with Experimental Data: Toward Practical Accuracy

This section presents the performance of the fine-tuned TeNNet-SAC model. During fine-tuning, all model components and layers were frozen except for the output head of the pretrained Γ predictor, allowing the model to adjust to experimental data with minimal changes to the learned representations. To assess robustness and generalization, we performed fine-tuning across 10 different random data splits, each defined by a unique seed. Table 1 summarizes the test set prediction errors, including mean absolute error (MAE) and mean squared error (MSE), for the TeNNet-SAC base model, the COSMO-SAC model, and the fine-tuned TeNNet-SAC model. The results show that fine-tuning consistently improves performance across all metrics. On average, the fine-tuned model achieves the lowest MAE (0.098) and MSE (0.058), outperforming both the base model (MAE = 0.154, MSE = 0.098) and COSMO-SAC (MAE = 0.122, MSE = 0.063).

**Table 1:** Test Set Prediction Errors for Activity Coefficients (ln γ) Using the TeNNet-SAC Base Model, COSMO-SAC, and the Fine-Tuned TeNNet-SAC Model^a^

	MAE			MSE		
seed	TeNNet-SAC_Base_	COSMO-SAC	TeNNet-SAC_Fine‑tuned_	TeNNet-SAC_Base_	COSMO-SAC	TeNNet-SAC_Fine‑tuned_
1	0.146	0.095	0.086	0.071	0.029	0.026
2	0.169	0.108	0.091	0.131	0.053	0.047
3	0.168	0.123	0.089	0.083	0.042	0.035
4	0.152	0.139	0.091	0.093	0.088	0.049
5	0.113	0.138	0.118	0.047	0.073	0.060
6	0.154	0.128	0.123	0.119	0.088	0.106
7	0.185	0.098	0.125	0.172	0.075	0.099
8	0.127	0.110	0.078	0.067	0.051	0.044
9	0.184	0.141	0.119	0.139	0.081	0.098
10	0.142	0.137	0.065	0.051	0.049	0.016
average	0.154	0.122	**0.098**	0.098	0.063	**0.058**
STD	0.022	0.017	**0.020**	0.039	0.020	**0.030**

a Each row corresponds to a different random seed used to split the dataset. The last two rows show the average and standard deviation (STD) over 10 seeds.

For practical applications, we recommend using the ensemble mean of the 10 fine-tuned models, referred to as the TeNNet-SAC fine-tuned ensemble model, to predict molecular activity coefficients. This ensemble approach maximizes reliability and provides a straightforward method for assessing prediction uncertainty. Figure 11 illustrates the distribution of absolute errors (MAE) between predicted and experimental ln γ values across all binary systems in the *D*_EXP_ dataset. The TeNNet-SAC fine-tuned ensemble model and COSMO-SAC both achieve overall coverage, with 98.5% of predictions falling within the [0, 0.5] MAE range. However, the fine-tuned ensemble model’s error distribution is more sharply concentrated at lower MAE values, as shown by the taller blue bars in the low-error region and a steeper cumulative distribution curve. This indicates that the TeNNet-SAC fine-tuned ensemble model not only matches COSMO-SAC in broad accuracy metrics but can tend to produce fewer high-error outliers, reflecting its ability to learn from experimental data and correct for input descriptor uncertainties. In contrast, the TeNNet-SAC base model shows a broader distribution with a slightly lower proportion (96.0%), underscoring the value of the fine-tuning step in achieving robust, low-error predictions from SMILES-based inputs.

**Figure 11:**
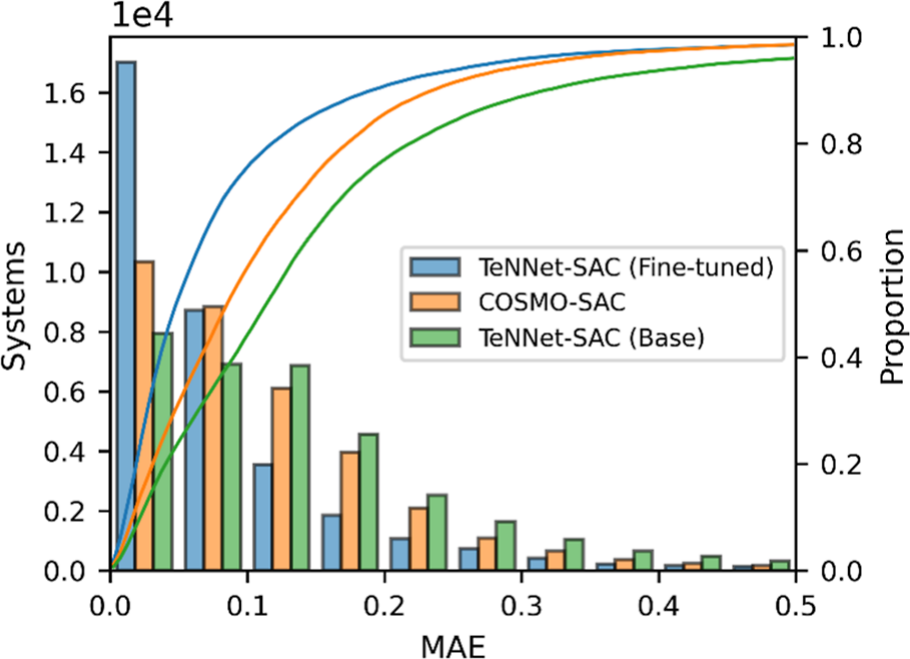
Distribution of absolute errors (MAE) between predicted and experimental ln γ values for binary systems. Blue bars show results from the TeNNet-SAC fine-tuned model, orange bars represent predictions from the COSMO-SAC model, and green bars correspond to the TeNNet-SAC base model. Lines show the cumulative distribution functions (CDFs), reaching 0.985 (blue), 0.985 (orange), and 0.960 (green) within the plotted range.

Table 2 compares the performance of different methods for the three types of mixtures: the HB, polar (no-HB), and weak/nonpolar categorized in Section 2.2.3. The results indicate that the TeNNet-SAC fine-tuned ensemble model consistently achieves lower MAE and MSE values than COSMO-SAC across all categories, particularly for hydrogen-bonding systems where it reduces the MAE from 0.134 to 0.098. This suggests an improved capacity to capture strong associative interactions when sufficient experimental data are used during fine-tuning. Additionally, error levels vary substantially across categories for all models, with generally lower errors observed for weak/nonpolar systems, showing that simpler, less interactive mixtures are easier to predict accurately.

**Table 2:** All Dataset Prediction Errors for Activity Coefficients (ln γ) Using the TeNNet-SAC Base Model, COSMO-SAC, and the Fine-Tuned TeNNet-SAC Ensemble Model^a^

			TeNNet-SAC_Fine‑tuned_		COSMO-SAC		TeNNet-SAC_Base_	
data type	#mixture	#data	MAE	MSE	MAE	MSE	MAE	MSE
HB	282	26,479	0.098	0.053	0.134	0.067	0.183	0.119
polar, no HB	68	3182	0.083	0.022	0.099	0.032	0.107	0.039
weak/nonpolar	47	4710	0.036	0.005	0.049	0.009	0.049	0.009
total	397	34,371	0.088	0.043	0.119	0.056	0.157	0.097

a HB: systems containing hydrogen-bonding compounds; polar, no-HB: systems with strong polarity but without hydrogen-bonding functionality; weak/nonpolar: systems that are weakly polar or essentially nonpolar.

Figure 12 demonstrates representative predictions for five selected binary systems, consistent with those shown in Figure 10. The uncertainties shown as blue shades are calculated from the standard deviation of the outputs of the 10 fine-tuned models (Table 1). These examples illustrate how the TeNNet-SAC framework improves its accuracy from the COSMO-SAC level toward a more practical, experimentally aligned level. In all cases, the fine-tuned TeNNet-SAC ensemble model outperforms COSMO-SAC, demonstrating enhanced predictive capability after fine-tuning. Notably, this improvement is achieved by fine-tuning only the output head of the Γ predictor, highlighting the strong transferability of the pretrained representation.

**Figure 12:**
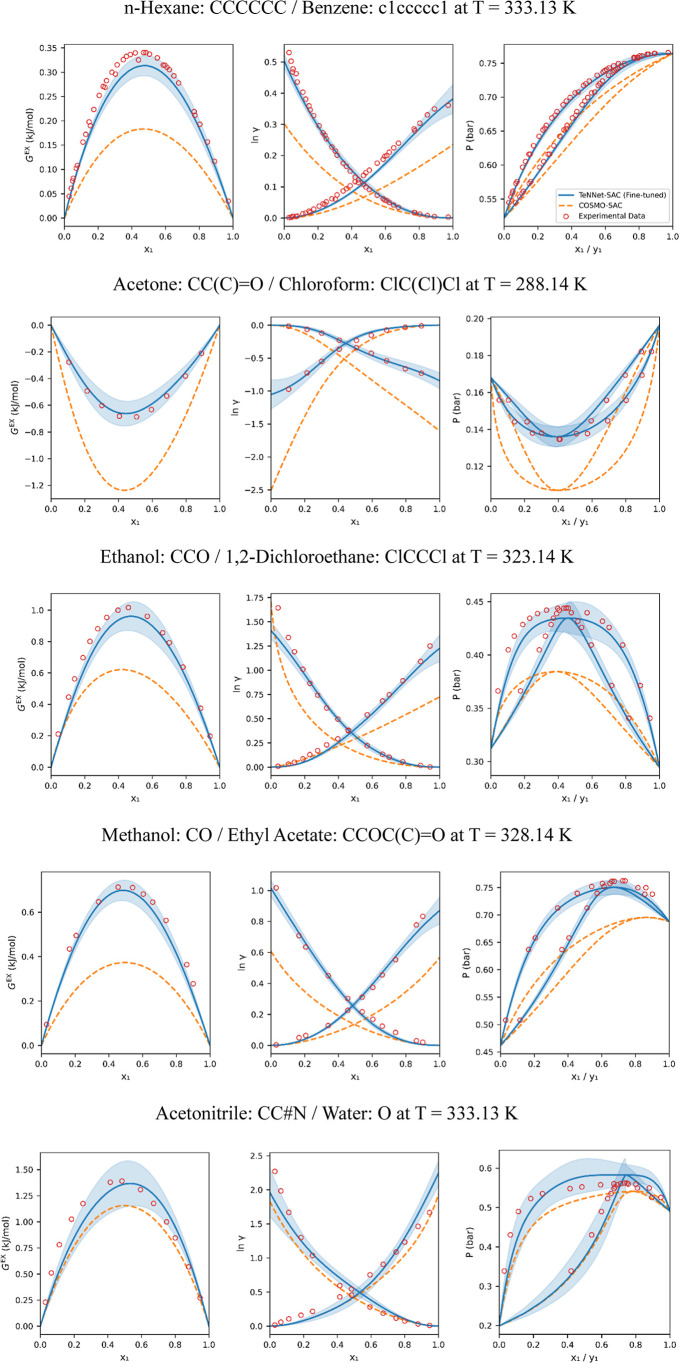
From left to right: predicted excess Gibbs free energies (*G* ^ex^), logarithmic activity coefficients (ln γ), and isothermal vapor–liquid equilibrium diagrams for binary systems. The TeNNet-SAC fine-tuned model predictions are shown as blue lines, with light-blue shading indicating the ensemble standard deviation. COSMO-SAC results are represented by orange dashed lines, and experimental data are plotted as red circles.

## 4 Conclusions

Efficient and accurate prediction of activity coefficients in multicomponent systems poses a longstanding challenge in solution thermodynamics, particularly due to the limitations of existing models and the scarcity of experimental data. In this work, we propose a physics-embedded machine learning framework, TeNNet-SAC, that leverages the conceptual foundation of COSMO-SAC to predict activity coefficients without relying on binary interaction parameters. This design enables broad applicability and scalability for complex fluid mixtures.

A central strength of TeNNet-SAC lies in its thermodynamic consistency. The Γ predictor is carefully designed to reflect fundamental principles of solution theory. The residual contribution, guided by COSMO-SAC, is embedded into the neural network, directly enforcing thermodynamic consistency. In parallel, the combinatorial term adopts the Staverman–Guggenheim formalism, with machine learning components embedded into the physical model, thereby enhancing both flexibility and theoretical rigor.

To improve generalization while avoiding overfitting, we employed a two-stage training strategy. In the pretraining stage, fully synthetic data we generated by first constructing artificial σ-profiles and then applying the COSMO-SAC model to compute the corresponding segment activity coefficients. This enabled the model to learn the general physical behavior and structure encoded in COSMO-SAC. Subsequently, fine-tuning was performed using limited experimental data. This approach not only enhanced accuracy but also prevented catastrophic forgetting, maintaining consistency between the base and fine-tuned models across diverse mixtures.

Finally, the modular nature of TeNNet-SAC allows flexibility in input features. For instance, the Γ predictor can be directly applied using real σ-profiles obtained from quantum chemical calculations, enabling seamless integration with other computational chemistry workflows. Taken together, TeNNet-SAC offers a physically grounded and generalizable approach for predicting activity coefficients in complex mixtures.

## Supplementary Material



## Data Availability

The GitHub repository https://github.com/yueyue2299/TeNNet-SAC provides the following trained models: (1) the σ-profile predictor and geometry predictor, both trained with a 90%/10% train/validation split without a separate test set. (2) The base model used prior to fine-tuning. (3) The fine-tuned model, provided as an ensemble constructed by averaging the predictions of 10 models trained with different random seeds (as listed in Table 1).

## Notes

Methods for generating artificial σ-profiles, Aspen Plus quality scoring, hyperparameter settings and loss functions, definitions of error metrics, additional prediction matrices, computational details, and proof of thermodynamic consistency of TeNNeT-SAC Parameter ranges for Gaussian distributions in the simulation of σ-profiles (Table S1), performance metrics for the geometry predictor (Tables S2–S4), discussion of charge neutrality in σ-profiles (Figures S1–S3, Tables S5–S6), computational efficiency of Γ-predictor (Table S7), COSMO-SAC parameter values (Table S8), and a representative σ-profile of water (Figure S4) (PDF)
