# Supplementary material for: Physics-Embedded Machine Learning Model for Phase Equilibrium Prediction in Multicomponent Systems
Source: J Chem Inf Model. 2025 Sep 22;65(19):10180–93. doi: 10.1021/acs.jcim.5c01804 (PMC12529776; doi:10.1021/acs.jcim.5c01804)
Supplement: Supplementary file 1 [file ci5c01804_si_001.pdf]

Supporting Information for

Physics-Embedded Machine Learning Model for Phase Equilibrium Prediction in Multicomponent Systems

Yue Yang and Shiang-Tai Lin*

Department of Chemical Engineering, National Taiwan University, Taipei, 106319, Taiwan

S1. Settings for Artificial Profile Generation

In this work, we generate random Gaussian-superimposed curves to simulate σ-profile. The overall profile $p(\sigma_j)$ is constructed by superimposing multiple Gaussian distributions, as shown in eq (S1). The σ spans from -0.05 to 0.05 with 101 discrete points, as shown in eq (S2). The resulting profile is then processed to yield the final scaled form $\bar{p}(\sigma_j)$ used in this study.

$$p(\sigma_j) = \sum_{m=1}^{3}\sum_{i=1}^{I} A_{mi}\exp\left(-\frac{(\sigma_j - \sigma_{mi})^2}{2\delta_{mi}^2}\right) \quad \text{(S1)}$$

$$\sigma_i = \frac{i}{1000}, \qquad i \in \{-50, \cdots, 50\} \quad \text{(S2)}$$

where, $p(\sigma_j)$: The height of the profile at σ_j. σ_{mi}: The mean position of the i-th Gaussian distribution. δ_{mi}: The half-width of the i-th Gaussian distribution. A_{mi}: The amplitude of the i-th Gaussian distribution. N_m: The number of Gaussian distributions.

The distribution parameters for each Gaussian group m are defined as Table S1.

Table S1 Parameter ranges for Gaussian distributions in the simulation of σ-profiles. Here, U[a,b] denotes a uniform random distribution between a and b.

m	N_m	σ_{mi}	δ_{mi}	A_{mi}
1	U{5,…, 11}	25% probability each: U[-0.016,0], U[0,0.016], U[-0.01,0.01], or 0	U[0.0002,0.001]	U[0.1,30]
2	U{4,…, 17}	U[-0.003,0.003]	U[0.0002,0.0025]	U[0.1,100]
3	10% probability of inclusion, N = 30; otherwise N = 0	U[-0.02,0.02]	U[0.0002,0.001]	U[0.1,100]

The profile is then truncated at extreme positions, setting values to zero outside the range $-0.025 < \sigma_j < 0.025$, as shown in eq (S3). Finally, the truncated profile $\tilde{p}(\sigma_j)$ is scaled to match a specified area, where *area* = U[20,1600] as shown in eq (S4).

$$\tilde{p}(\sigma_j) \leftarrow \begin{cases} 0, & \sigma_j < -0.025 \ \ or \ \ \sigma_j > 0.025 \\ p(\sigma_j), & \text{otherwiae} \end{cases} \tag{S3}$$

$$\bar{p}(\sigma_j) = \tilde{p}(\sigma_j)\frac{area}{\sum_{k=0}^{100}\tilde{p}(\sigma_k)} \tag{S4}$$

S2. Aspen Plus Quality Scoring

A quality score threshold of 0.25 was adopted to retain only reliable data points for training. Since most consistency tests in Aspen Plus assign scores ranging from 0.1 to 1.0, a score above 0.25 typically indicates that (i) multiple consistency tests could be successfully applied, or (ii) the available tests, such as the infinite dilution test, yielded non-minimal quality factors. This threshold therefore effectively filters out entries that are either incomplete or significantly inconsistent with thermodynamic constraints. The following sections describe each of these consistency tests. This scoring criterion is primarily based on guidelines provided in the Aspen Plus V12 Help documentation[1] and supported by a relevant study[2].

To ensure thermodynamic reliability, each VLE dataset in Aspen Plus is evaluated using a series of consistency tests derived from the Gibbs–Duhem equation. These tests assess whether the data satisfy known thermodynamic constraints on the excess Gibbs energy and its derivatives with respect to pressure, temperature, and composition. The overall VLE quality score is calculated by averaging the quality factors $F_{test,j}$ of all applicable tests, and multiplying by the quality factor F_{pure} as shown in eq (S5), where

N is the number of applicable consistency tests. If no test is applicable, the default quality score is reduced to eq (S6).

$$q_n = F_{pure} \frac{\sum_j F_{test,j}}{N} \tag{S5}$$

$$q_n = 0.5F_{pure} \tag{S6}$$

Endpoint consistency test

The Endpoint Consistency Test verifies whether the vapor pressures at the composition limits of the VLE curve are consistent with the pure component vapor pressures. When a binary mixture approaches a pure component, the total pressure should approach the vapor pressure of the corresponding pure substance. This check ensures that the experimental or extrapolated VLE data agrees with known pure component properties. For isothermal T-p-x data, the test applies to the bubble pressure and for T-p-y data, the corresponding dew point expressions, as shown in eq (S7).

$$p_{bubble} = \begin{cases} p_1^0, & x_1 \to 1 \\ p_2^0, & x_1 \to 0 \end{cases} \qquad p_{dew} = \begin{cases} p_1^0, & y_1 \to 1 \\ p_2^0, & y_1 \to 0 \end{cases} \tag{S7}$$

The consistency is quantified by the relative deviation between the bubble (or dew) pressure and the known pure component vapor pressure. For component i, this is

expressed as eq (S8). If both deviations for component 1 and 2 are below 1%, the test is considered passed. Otherwise, a quality factor F_{pure} is calculated as eq (S9). The resulting consistency factor F_{pure} ranges from 0.1 (poor agreement) to 1.0 (perfect agreement).

$$\Delta p_i^0 = \left|\frac{p_{bubble}(x_i \to 1) - p_i^0}{p_i^0}\right| \quad \text{(S8)}$$

$$F_{pure} = \frac{2}{100(\Delta p_1^0 + \Delta p_2^0)} \quad \text{(S9)}$$

Herington test

The Herington Test evaluates the thermodynamic consistency of VLE data by checking compliance with the Gibbs–Duhem equation over the entire composition range. It is based on the following integrated form of the Gibbs–Duhem relation, as shown in eq (S10). For isothermal or isobaric conditions, this relation simplifies to the expressions given in eq (S11) and eq (S12).

$$\sum_i x_i dln\ \gamma_i - \frac{V^E}{RT}dp + \frac{H^E}{RT^2}dT = 0 \quad \text{(S10)}$$

$$A^* = 100\left(\int_0^1 ln\ \frac{\gamma_1}{\gamma_2}dx + \int_0^1 \varepsilon dx\right) \quad \text{(S11)}$$

$$\varepsilon = \begin{cases} \left(\frac{V^E}{RT}\right)\left(\frac{\partial p}{\partial x_1}\right)_T, & \text{(isothermal)} \\ \left(\frac{H^E}{RT^2}\right)\left(\frac{\partial T}{\partial x_1}\right)_p, & \text{(isobaric)} \end{cases} \quad \text{(S12)}$$

Wisniak's modification[3] of the original Herington method is used, introducing two indicators: the symmetry deviation, $D = 100\left|\frac{A-B}{A+B}\right|$, and the temperature range factor, $J = 150\left|\frac{\Delta T_{max}}{T_{min}}\right|$. Here, A represents the area where $ln\ \frac{\gamma_1}{\gamma_2}$ lines above zero across x, while B is the area where it lies below zero.

The test is considered passed if $|A^*| < 3$. Otherwise, a quality factor $F_{test,1}$ is assigned based on the degree of inconsistency as shown in eq (S13). The resulting value ranges from 0.1 (poor consistency) to 1.0 (perfect consistency), and is incorporated into the overall VLE data quality evaluation.

$$F_{test,1} = \begin{cases} 0.25 \times \frac{5}{D}, & \text{(isothermal)} \\ \frac{10}{D-J}, & \text{(isobaric)} \end{cases} \quad \text{(S13)}$$

Van Ness test

The Van Ness test evaluates the ability of a mathematical activity coefficient model to reproduce experimental VLE data. In this study, the five-parameter NRTL model is employed to predict bubble pressures and vapor-phase compositions using nonlinear

regression of experimental T–x–y or T–p–x data.

The model parameters are fitted to minimize the deviation between experimental and calculated properties. For isothermal datasets, binary interaction parameters are assumed to be composition-dependent, while for isobaric data they are modeled as temperature-dependent. The fitted model is assessed against two performance criteria, as shown in eq (S14) and eq (S15).

$$\Delta p = \frac{1}{N}\sum_{i=1}^{N} 100\left|\frac{p_i^{exp} - p_i^{cal}}{p_i^{exp}}\right| \tag{S14}$$

$$\Delta y = \frac{1}{N}\sum_{i=1}^{N} 100\left|y_i^{exp} - y_i^{cal}\right| \tag{S15}$$

If both Δp and Δy are less than 1, the dataset is considered to pass the Van Ness test. Otherwise, a quality factor is assigned based on the total deviation as shown in eq (S16). The resulting value ranges from 0.1 (poor consistency) to 1.0 (perfect consistency).

$$F_{test,2} = \frac{2}{(\Delta p + \Delta y)} \tag{S16}$$

Point test

The point test evaluates thermodynamic consistency at individual composition points in isothermal VLE datasets. It is based on the differential form of the Gibbs–Duhem equation and compares the slope of the excess Gibbs energy with the logarithmic activity coefficient difference. The overall deviation is calculated as eq (S17).

$$\delta_k = \left(\frac{d(G^E/RT)}{dx_1} - ln\ \frac{\gamma_1}{\gamma_2} - \varepsilon\right) \qquad \text{(S17)}$$

The overall deviation is calculated as $\delta = 100\sum_{k=1}^{N} \delta_k/N$. If $\delta < 5$, the dataset is considered consistent. Otherwise, a quality factor is assigned as eq (S18). The resulting value ranges from 0.1 (poor consistency) to 1.0 (perfect consistency).

$$F_{test,3} = \frac{5}{\delta} \qquad \text{(S18)}$$

Infinite dilution test

The infinite dilution test examines the limiting behavior of $G^E/(x_1x_2RT)$ as one component approaches zero concentration. The test checks whether the limiting behavior of the excess Gibbs energy is consistent with the activity coefficient ratios. The percentage deviations at each infinite dilution limit are computed as eq (S19). If

both $I_1 < 30$ and $I_2 < 30$, the dataset is considered consistent. Otherwise, the following quality factor is applied as eq (S20). The resulting quality factor is limited to a maximum of 0.25 and reflects the consistency of the model in capturing limiting behavior.

$$I_j = 100 \left| \frac{\frac{G^E}{x_1 x_2 RT} - ln \frac{\gamma_1}{\gamma_2}}{ln \frac{\gamma_1}{\gamma_2}} \right|_{x_j=0} \quad \text{(S19)}$$

$$F_{test,4} = \frac{15}{I_1 + I_2} \quad \text{(S20)}$$

EOS test

The EOS test evaluates the performance of an equation of state model in predicting high-pressure VLE data. The Peng–Robinson equation of state[4] is combined with the UNIQUAC activity coefficient model[5] through the Universal Mixing Rule (UMR) proposed by Voutsas et al.[2] This test is applied exclusively to T–P–x–y datasets with pressures above 1 MPa.

The EOS model is fitted to the experimental data by minimizing the deviation in pressure and vapor-phase composition. Regions near the critical point are excluded due to known model limitations. After fitting, the consistency is evaluated using the same deviation metrics as in the Van Ness test.

If both Δp and Δy are less than 1, the dataset is considered to pass the EOS test. Otherwise, a quality factor is calculated as eq (S21). The resulting value ranges from 0.1 (poor consistency) to 1.0 (perfect consistency)

$$F_{test,5} = \frac{2}{(\Delta p + \Delta y)} \tag{S21}$$

S3. Hyperparameter Settings and Loss Functions

All model training and evaluation were conducted on Google Colab using an NVIDIA T4 GPU. Model checkpoints were saved in .ckpt format for reproducibility.

Base model

Each of the three components—the σ-profile predictor, geometry predictor, and Γ predictor—was trained independently using early stopping based on validation loss. All models were initialized with a random seed of 1. The σ-profile and geometry predictors were trained for up to 1600 epochs with a batch size of 128, and early stopping was triggered after 400 epochs without validation improvement. The Γ predictor was trained for up to 240 epochs with a batch size of 512, and early stopping was applied with a patience of 60 epochs. To stabilize training and avoid overfitting, ReduceLROnPlateau learning rate schedulers were employed in all three models. Additionally, dropout

regularization was applied to the output layers of the σ-profile predictor to improve generalization, with a default dropout rate of 0.25 and one layer set to 0.2.

Each component model was trained using a distinct optimization configuration tailored to its task. The σ-profile predictor employed the AdamW optimizer with a weight decay of 1×10^{-5} and an initial learning rate of 3.2×10^{-4}. The loss function used was mean squared error (MSE), and a ReduceLROnPlateau scheduler was applied, reducing the learning rate by a factor of 0.8 if the validation loss did not improve for 24 consecutive epochs. The geometry predictor was trained using the Adam optimizer with an initial learning rate of 3.2×10^{-4} and no weight decay. The loss function was the mean absolute percentage error (MAPE), where $\varepsilon = 1 \times 10^{-8}$ is a small constant used to prevent division by zero. A learning rate scheduler reduced the learning rate by a factor of 0.5 after 20 epochs without validation improvement.

$$\mathcal{L}_{MAPE} = \frac{1}{N}\sum_{i=1}^{N}\left|\frac{y_i - \hat{y}_i}{y_i + \varepsilon}\right| \tag{S22}$$

For the Γ predictor, the Adam optimizer with a weight decay of 1.0×10^{-5} and a learning rate of 1.0×10^{-3} was used. The loss function combined mean absolute error (MAE) with L1 and L2 regularization terms, each with a regularization coefficient λ of 1.0×10^{-5}. A ReduceLROnPlateau scheduler with a factor of 0.5 and a patience of 5

epochs was employed to adjust the learning rate adaptively. All hyperparameters were selected empirically based on validation performance and were found to yield stable convergence across runs.

Fine-tuned model

During fine-tuning, only the parameters of the output head were updated, while the rest of the network remained frozen. The loss function was the mean squared error (MSE), with each sample weighted by the square of its associated data quality score, where q_i denotes the quality score of the i-th data point.

$$\mathcal{L}_{fine-tuning} = \frac{1}{N}\sum_{i=1}^{N} q_i^2 (y_i - \hat{y}_i)^2 \tag{S23}$$

Fine-tuning was performed using the Adam optimizer with a weight decay of 1.0×10^{-5}. The learning rate followed a warm-up cosine annealing schedule: it increased linearly to a maximum of 1.6×10^{-4} over the first 10 epochs, and then gradually decayed toward a minimum of 1.0×10^{-6} following the cosine function, where T is the total number of training epochs and T_{warmup} is number of warm-up epochs. Training was conducted for up to 200 epochs with a batch size of 32. Early stopping was triggered if the validation loss failed to improve by at least 2.0×10^{-4} over 40 consecutive epochs.

$$\mathrm{lr}(t) = \begin{cases} lr_{max}\dfrac{t+1}{T_{warmup}}, t < T_{warmup} \\ lr_{max} + \dfrac{1}{2}(lr_{max} - lr_{min})\left(1 + cos\left(\dfrac{\pi(t - T_{warmup})}{T}\right)\right), otherwise \end{cases} \quad \text{(S24)}$$

S4. Error Metrics Definitions

This appendix describes the error metrics used to evaluate the predictive performance of the models for different types of properties. For scalar properties such as area, volume, and ln γ, the evaluation was based on the mean absolute error (MAE), mean squared error (MSE), percentage mean absolute error (%MAE), and the coefficient of determination (R^2). The corresponding mathematical definitions are given by

$MAE = \frac{1}{N}\sum_{i=1}^{N}\lvert y_i - \hat{y}_i\rvert$	(S25)
$MSE = \frac{1}{N}\sum_{i=1}^{N}(y_i - \hat{y}_i)^2$	(S26)
$\%MAE = \frac{1}{N}\sum_{i=1}^{N}\left\lvert\frac{y_i - \hat{y}_i}{y_i}\right\rvert$	(S27)
$R^2 = 1 - \frac{\sum_{i=1}^{N}(y_i - \hat{y}_i)^2}{\sum_{i=1}^{N}(y_i - \bar{y})^2}$	(S28)

where N is the number of data point and $\bar{y} = \frac{1}{N}\sum_{i=1}^{N} y_i$ is the mean of the true values.

For the σ-profile predictions, which consist of multi-dimensional segment data, the metrics were generalized to account for the additional dimensions. The errors were averaged over all segments and all samples. The mean absolute error (MAE) and root mean squared error (RMSE) for σ-profile predictions were defined as the average absolute and squared errors across all segments and samples, while the coefficient of determination (R^2) measures how well the predicted profiles capture the variance in the true profiles. The corresponding formulas are

$MAE = \frac{1}{N}\sum_{i=1}^{N}\frac{1}{n}\sum_{j=1}^{n}\left\|y_{i,j} - \hat{y}_{i,j}\right\|$	(S29)
$RMSE = \sqrt{\frac{1}{N}\sum_{i=1}^{N}\frac{1}{n}\sum_{j=1}^{n}\left(y_{i,j} - \hat{y}_{i,j}\right)^2}$	(S30)
$R^2 = 1 - \frac{\sum_{i=1}^{N}\sum_{j=1}^{n}\left(y_{i,j} - \hat{y}_{i,j}\right)^2}{\sum_{i=1}^{N}\sum_{j=1}^{n}\left(y_{i,j} - \bar{y}_j\right)^2}$	(S31)

where n is the number of segments, N is the number of data, and $\bar{y} = \frac{1}{N}\sum_{i=1}^{N} y_{i,j}$ denotes the mean value for segment j across all samples.

S5. Additional Prediction Matrices

Table S2. Performance metrics for the geometry predictor on molecular surface area across training, validation, and test sets.

Dataset	Sample Count	MAE	RMSE	R^2	% MAE
Train	31203	2.683	4.717	0.998	0.89%
Valid	3903	3.564	5.566	0.997	1.21%
Test	3901	3.771	6.040	0.997	1.24%

Table S3. Performance metrics for the geometry predictor on molecular volume across training, validation, and test sets.

Dataset	Sample Count	MAE	RMSE	R^2	% MAE
Train	31203	1.975	3.291	0.999	0.60%
Valid	3903	2.837	4.328	0.999	0.93%
Test	3901	2.849	4.296	0.999	0.91%

Table S4. Area-scaled σ-profile prediction performance across training, validation, and test sets.

Dataset	Sample Count	MAE	RMSE	R^2
Train	31203	0.440	0.767	0.989
Valid	3903	0.782	1.453	0.959
Test	3901	0.779	1.448	0.962

S6. Charge Neutrality of σ-profiles

In the σ-profile predictor presented in this work, charge neutrality is not explicitly enforced. As a result, the predicted profiles exhibit charge distributions that fluctuate around zero, as illustrated in Figure S1. Our analysis indicates that enforcing strict charge neutrality does not necessarily reduce the overall prediction error. On the contrary, it can compromise the accuracy in the polar region of the σ-profile, which, although relatively small in magnitude, is highly sensitive and plays a critical role in activity coefficient predictions. Because both COSMO-SAC and the Γ predictor rely heavily on capturing accurate features in the polar region, preserving the intrinsic σ-profile patterns is more important for predictive performance than enforcing neutrality. To further validate this conclusion, we investigated two approaches for addressing charge neutrality:

1. Soft constraint – introducing a penalty term in the training loss function of the σ-profile predictor to encourage charge neutrality.

2. Hard constraint – rescaling the surface area of each σ-profile such that the total charge integrates to zero.

The resulting charge distributions under these two treatments are shown in Figures S2 and S3, respectively.

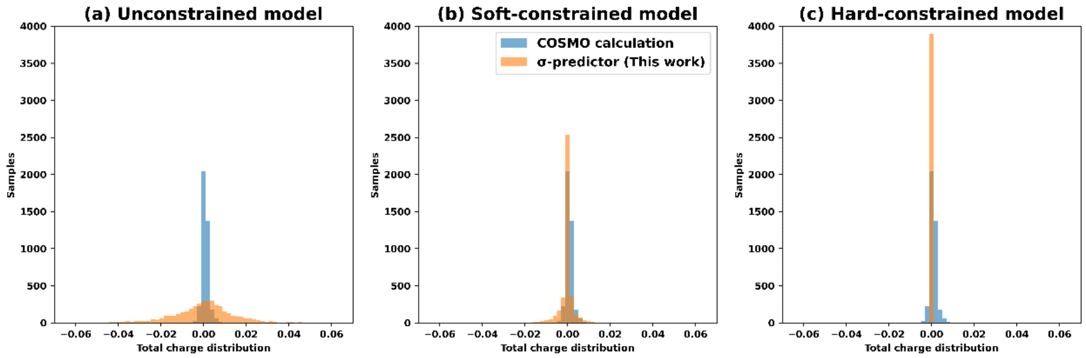

Figure S1. Distribution of molecular net charge calculated from the σ-profile predictor (a) without constraints on the charge neutrality, (b) with charge neutrality soft-constrained in the loss function, and (c) with rescaling of the σ-profile to ensure charge neutrality. The distribution of molecular net charge from the σ-profile derived from COSMO calculation is shown in blue.

Table S5 summarizes the predictive performance of the three σ-profile predictors on the test set. The results show that the unconstrained and soft-constrained models perform nearly identically, while the hard-constrained model exhibits slightly inferior accuracy. At this point, one might expect the soft-constrained model to be the most favorable choice, as it better approximates charge neutrality without sacrificing accuracy. However, our primary concern is not only the accuracy of σ-profile prediction, but also the resulting performance in activity coefficient predictions. Table S6 reports the results when the three σ-profile predictors are used as inputs to the Γ-predictor (evaluated on

the same dataset as in Figure 9). Here, both the soft- and hard-constrained models perform worse than the unconstrained baseline.

For the hard-constrained model, the decline in activity coefficient accuracy can be attributed to its reduced ability to reproduce σ-profiles. The case of the soft-constrained model is more subtle. The inclusion of a charge-penalty term during training biases the model toward generating fewer charged segments, since reducing the number of charged segments makes it easier to satisfy charge neutrality. This bias leads to increased errors in the polar region of the σ-profile. While the soft-constrained model achieves similar overall accuracy by compensating with improvements in the nonpolar regions, the polar region is precisely the most critical factor for accurate activity coefficient predictions. Thus, enforcing charge neutrality, although physically appealing, ultimately degrades the predictive power for activity coefficients.

Nevertheless, we emphasize that ensuring charge correctness remains scientifically valuable. Our future efforts will focus on developing approaches that preserve the charge characteristics of σ-profiles (including both neutral and ionic species) while maintaining the accuracy of activity coefficient predictions.

Table S5. Performance of three σ-profile predictors on the test set.

Model	MAE	RMSE	R^2
Unconstrained (This work)	0.7793	1.448	0.9619

Soft-constrained	0.7880	1.449	0.9613
Hard-constrained	0.8184	1.565	0.9571

Table S6. Performance of activity coefficient predictions when using σ-profiles from the three predictors as inputs to the Γ-predictor (evaluated on the same dataset as in Figure 9).

Model	MAE	MSE
Unconstrained (This work)	0.0654	0.0403
Soft-constrained	0.0967	0.0655
Hard-constrained	0.0722	0.0520

S7. Computational Details

S7.1 Computational Efficiency of Γ-predictor

Here, we demonstrate the computational efficiency of both approaches by benchmarking the runtime for calculating activity coefficients in 10,000 binary systems. As summarized in Table S7, the NN Γ-predictor and the SAC component of COSMO-SAC exhibit comparable runtimes. The minor difference is attributable to the Python implementation of the NN compared with the C++ of COSMO-SAC. All computations were performed on a Linux workstation equipped with dual-socket AMD EPYC 9124 16-core processors (32 cores in total, 3.0 GHz) and 32 MB L3 cache per socket. All computations were performed on a Linux workstation equipped with dual-socket AMD

EPYC 9124 16-core processors (32 cores total, 3.0 GHz) and 32 MB L3 cache per socket.

Table S7. Benchmark of runtime for calculating activity coefficients of 10,000 binary systems using different implementations.

Model	Time used (sec)	Language	Device	Batch size
Γ-predictor (This work)	41.38	Python	CPU	1
SAC part of COSMO-SAC	28.18	C++	CPU	1
Γ-predictor (This work)	0.08465	Python	GPU	1024

S7.2 COSMO-SAC 2002 (ADF-optimized parameters)

The Γ-predictor was pretrained on synthetic data generated using COSMO-SAC 2002 model[6, 7]. In addition, this same COSMO-SAC model was employed as a reference for subsequent performance comparison with our work. The corresponding parameter values are summarized in Table S8.

Table S8. Values of parameter in COSMO-SAC 2002 (ADF)

f_{pol}	C_{hb} (kcal Å^4/mol e^2)	σ_{HB} (e/Å^2)	a_{eff} (Å^2)	f_{decay}	r (Å^2)	q (Å^2)
0.7167	100,664	0.0084	5.8447	3.5722	66.69	79.53

S7.3 σ-profile of water

As a representative and commonly used solvent, water is shown here as an example. Figure S2 compares the predicted and reference σ-profiles, with corresponding performance metrics of MSE = 0.1252, MAE = 0.2377, and R^2 = 0.8015.

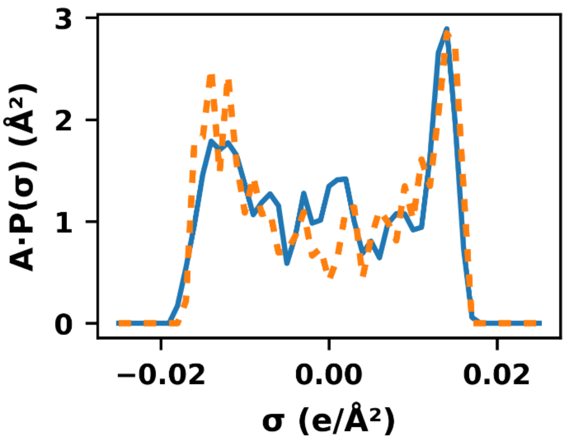

Figure S2. σ-profile of water. The blue line represents the predicted profile; the orange dashed line shows the true profile.

S8. Proof of Thermodynamic Consistency of TeNNet-SAC

The following derivation shows that the residual term of the molecular activity coefficient also satisfies the Gibbs-Duhem relation when the segment activity coefficients are consistent. From eq (9), the Gibbs-Duhem relation expressed at segment level is

$\sum_{\sigma_m} n_i(\sigma_m) d\ln\ \Gamma_i(\sigma_m) = 0$	(S32)

Based on eq (5), the total differential of the residual term of the molecular activity coefficient can be written as

$d\ln\ \gamma_{i/S}^{res} = n_i \left\{ \sum_{\sigma_m} dp_i(\sigma_m)[\ln\ \Gamma_S(\sigma_m) - \ln\ \Gamma_i(\sigma_m)] + \sum_{\sigma_m} p_i(\sigma_m)[d\ln\ \Gamma_S(\sigma_m) - d\ln\ \Gamma_i(\sigma_m)] \right\}$	(S33)

Here, $dp_i(\sigma_m) = 0$, and the term $-n_i \sum_{\sigma_m} p_i(\sigma_m) d\ln\ \Gamma_i(\sigma_m) = 0$ by eq (S32). The expression thus reduces to

$d\ln\ \gamma_{i/S}^{res} = n_i \sum_{\sigma_m} p_i(\sigma_m) d\ln\ \Gamma_S(\sigma_m)$	(S34)

Returning to the Gibbs-Duhem relation at the molecular level, and by combining eq (S32) and eq (S34), it follows that the molecular residual activity coefficients also satisfy thermodynamic consistency:

$\sum_i N_i\, d\ln\ \gamma_{i/S}^{res} = \sum_i N_i n_i \sum_{\sigma_m} p_i(\sigma_m) d\ln\ \Gamma_S(\sigma_m) = N \sum_{\sigma_m} \sum_i x_i\, n_i(\sigma_m)\, d\ln\ \Gamma_S(\sigma_m) = N \sum_{\sigma_m} n_S(\sigma_m)\, d\ln\ \Gamma_S(\sigma_m) = 0$	(S35)

Thus, thermodynamic consistency is rigorously preserved from the segment level to the molecular level.

REFERENCE

1. I. Aspen Technology, *Journal*, 2003, Computer software.
2. E. Voutsas, V. Louli, C. Boukouvalas, K. Magoulas and D. Tassios, *Fluid Phase Equilibria*, 2006, **241**, 216-228.
3. J. Wisniak, *Industrial & engineering chemistry research*, 1994, **33**, 177-180.
4. D.-Y. Peng and D. B. Robinson, *Industrial & Engineering Chemistry Fundamentals*, 1976, **15**, 59-64.
5. D. S. Abrams and J. M. Prausnitz, *AIChE journal*, 1975, **21**, 116-128.
6. C.-K. Chang, W.-L. Chen, D. T. Wu and S.-T. Lin, *Industrial & Engineering Chemistry Research*, 2018, **57**, 11229-11238.
7. S. T. Lin and S. I. Sandler, *Industrial & Engineering Chemistry Research*, 2002, **41**, 899-913.
